# Editorial: Decompressive Craniectomy and Cranioplasty - Challenges and Chances

**DOI:** 10.3389/fsurg.2022.945290

**Published:** 2022-06-09

**Authors:** Ciaran Scott Hill, Christian Henker, Julius Höhne

**Affiliations:** ^1^ National Hospital for Neurology and Neurosurgery (NHNN), London, United Kingdom; ^2^ Department of Neurosurgery, University Hospital Rostock, Rostock, Mecklenburg-Vorpommern, Germany; ^3^ Department of Neurosurgery, University Medical Center Regensburg, Regensburg, Bavaria, Germany

**Keywords:** cranioplasty, decompression, neurosurgery, trauma, stroke, complication

**Editorial on the Research Topic**

***Decompressive Craniectomy and Cranioplasty - Challenges and Chances***

Cranioplasty is an ancient operation with the early records going back to the Incan empire in the 15th century. Surgery is predicated on the central tenants of ameliorating abnormal pathology and restoring defective anatomy (1). The Incan surgeon was surely compelled by the natural human inclination to fix that which appears broken. And what could be more convincing and primally ‘surgical’ than the need to repair a skull defect? However, even with such an apparently simple procedure- hidden complexities and obscure pitfalls abound. This themed Frontiers in Surgery issue addresses some of these challenges.

Firstly, in whom should the surgeon operate? While the controversy of decompressive craniectomies in trauma remain stubborn to any concluding argument, decompressions in the context of acute ischaemic stroke provide ample opportunities for those inclined towards cranioplasty insertion. The indications are important and require examination, not least in those without full capacity. Is this a cosmetic operation or a therapeutic one? Are we reducing future risk of injury? What are the neuro-cognitive implications of cranioplasty (or not performing cranioplasty)? And in what situations can we avoid cranioplasty altogether by replacing the bone at the time of primary surgery, for example after haematoma evacuation. These and many more questions pass through our minds during the process of consent. Our ability to weigh and balance these depends fundamentally upon research findings and our understanding of the data as applied to an individual patient. Using qualitative methods, Pandit et al. (2) investigate the question of whether there is a need for protection protocols in patients with craniectomy during non-ambulatory movements. Then adding to the conversation of patient selection and prognostication of surgery Lim et al. (3) present a multicentre study exploring intracranial pressure thresholds as a marker of adequacy in large territory ischaemic stroke.

Paediatric patients are a specific challenge. Their growing skulls require additional considerations. Bandyopadhyay (4) gives a brief overview of some key questions and concepts and the need for further research. An important question in cranioplasty is what material we should implant. While those early practitioners used a variety of precious metals and gourds, later practitioners have trialled autologous grafts, metals including titanium plates/meshes, ceramics, plastics, and latterly a variety of complex osteogenic materials. Augmentation with antibiotics have been used as have a plethora of structural variations. The work of Zaed et al. (5) to assesses outcomes in paediatric patients undergoing cranioplasty with custom-made porous hydroxyapatite plates is an example of how these questions need to be specifically addressed in children where adult findings may not immediately translate.

Next, how do we optimize the surgical implantation procedure itself. The examination of surgical workflows (including pre and postoperative aspects) and their impact is a vogue topic. He et al. (6) offer a perioperative paradigm and discuss how workflow can influence postoperative complications in the context of polyetheretherketone (PEEK) plates. Finally, the review by Mee et al. (7) gives a timely summary of the state of knowledge in the field, and show that despite being one of the first neurosurgical operations it is clear this procedure is still evolving and there remains significant room for refinement.
